# Effects of the COVID-19 Pandemic on Anger and Life Satisfaction among Children Aged 10-12 years old in Preveza

**DOI:** 10.1192/j.eurpsy.2024.1074

**Published:** 2024-08-27

**Authors:** D. Georgaki, C. Kalogirou, A. Potamianou, M. Bakola, P. Gourzis, G. Charalambous, E. Jelastopulu

**Affiliations:** ^1^Postgraduate Program Health Management, Federich University, Nicosia, Cyprus; ^2^Medical School, Department of Public Health; ^3^Medical School, Department of Pusychiatry, University of Patras, Patras, Greece

## Abstract

**Introduction:**

Children, who are particularly vulnerable in emergency situations, need tailored mental health strategies.

**Objectives:**

We investigated the impact of the COVID-19 pandemic on anger and life satisfaction in children.

**Methods:**

September 2021, we conducted a cross-sectional study in Preveza, Greece, interviewing 91 students aged 10-12 years from four elementary schools. The survey included socio-demographic questions, the Anger Expression Scale for Children (AESC), and the Satisfaction with Life Scale (SWLS). AESC scores range from 6 to 30 indicating anger severity, while SWLS scores between 5-9 signify extreme dissatisfaction and 31-35 extreme satisfaction.

**Results:**

Significant correlations were found between the number of siblings (p 0.004), duration of electronic play (p 0.005), and duration of sleep (p 0.014) with life satisfaction. Children without siblings, with limited play consumption, and early bedtimes had lower life satisfaction. The presence of a television in their room (p 0.027) and daily use of television and social media (p 0.007) correlated with anger management and behavior. Social media/TV use was associated with better anger management.

**Conclusions:**

Despite the pandemic lasting almost two years, children’s anger levels in Preveza remained stable, possibly due to outdoor activities and online interactions. These findings provide insights for policy makers, healthcare professionals, and parents seeking to improve anger management of children.

**Disclosure of Interest:**

None Declared

